# The Association Between Nurse Staffing and Conflict and Containment in Acute Mental Health Care: A Systematic Review

**DOI:** 10.1111/inm.70039

**Published:** 2025-04-07

**Authors:** Samuel Woodnutt, Simon Hall, Paula Libberton, Jane Ball, Chiara Dall'Ora, Peter Griffiths

**Affiliations:** ^1^ School of Health Sciences University of Southampton Hampshire UK; ^2^ The Royal College of Nursing London UK

**Keywords:** conflict and containment, incident reporting, inpatient mental health safety, mental health nurse staffing, patient safety, safer staffing, skill‐mix, staffing levels

## Abstract

Conflict and containment are the most frequently reported incidents in acute mental health care settings. This systematic review seeks to examine and synthesise existing evidence on the association between nurse staffing levels, nursing skill‐mix and the occurrence of these incidents in acute mental health wards. Systematic review of quantitative studies examining nurse staffing levels and skill‐mix (proportion of nursing shift that are registered or experience levels). Searches were undertaken in CINAHL, Cochrane, Embase, MEDLINE, PsycINFO, SCOPUS and Web of Science. Thirty‐five observational studies were reviewed, including 32 on staffing levels (44 analyses) and 12 on skill‐mix (14 analyses). Nine analyses found that higher staffing levels were associated with a reduction in reported conflict and containment incidents, while nine found lower staffing levels were associated with reduced incidents. Twenty‐six studies found no significant association. For skill‐mix, six analyses found that higher skill‐mix was associated with a reduction in incidents, seven found no significant association, while one analysis showed reduced skill‐mix was associated with a reduction in incidents. The results from analyses are mixed, with no clear conclusions on the relationship of staffing on incident rates. Studies often rely on routine or staff‐reported data that are prone to measurement and observer bias, where most analyses did not control for important factors, e.g., patient case‐mix or other patient‐related factors which could have influenced the results. Although higher staffing levels are sometimes associated with increased incident reporting, this may reflect greater interaction and reporting, or residual (unmeasured) confounding and/or lack of control for mediators and effect modifiers. The review highlights the need for better risk adjustment in observational studies, more refined methodologies and clearer definitions of outcomes to guide workforce planning and policy. Further large‐scale research is necessary to understand the complex relationships between staffing, skill‐mix and safety in mental health care. There is a major staffing crisis in mental health nursing, but evidence to understand the impact of this on patient outcomes and to guide staffing policies is missing, with several significant limitations in the existing evidence that need to be resolved. Identified evidence on mental health nurse staffing levels and skill‐mix is mixed and inconclusive; therefore, no clear implications for workforce planning or deployment can be recommended. However, this prompts debate on the nature and efficacy of routinely collected patient outcomes in clinical practice.

## 1 Introduction

Mental health inpatient safety is a key area of concern for global policy and research (World Health Organization [WHO] 2021). In England's National Health Service, incident reporting has doubled in recent years, where self‐harm and aggression contribute over 44% of all reported incidents (Woodnutt et al. 2024). The cause of the increase is unclear and may reflect increased reporting; nonetheless, these incidents are the most prevalent in mental health services globally (Staggs 2016; Weltens et al. 2021; Ngune et al. 2023) and therefore are an increasing demand on nursing time. Mental health nurses have crucial roles in the promotion of safety and prevention of behaviours that may harm others (e.g., violence, aggression and self‐injury) such as through preventative assessment and engagement and/or the promotion of emotional regulation (Hurley et al. 2022). However, there are significant global workforce deficits in mental health nursing (Adams et al. 2021).

The conflict and containment model conceptualises incidents in inpatient psychiatric settings (Bowers 2006) and is used to categorise patient safety outcomes (Pelto‐Piri et al. 2019; Ward‐Stockham et al. 2022) and inform guidance globally (Australian Capital Territory 2024; Council of Europe 2024). There are 17 conflict and 17 containment items in the original model (Bowers 2006) (see Table 1). Conflict refers to any incident that results in harm through interaction with other patients, staff and/or self‐injury and is currently the most commonly reported type of harm incident in most mental health settings (Staggs 2013; Woodnutt et al. 2024). Conflict includes incident sub‐types of aggression against others and against self (e.g., self‐harm or suicide attempts) but also behaviours such as refusal of medication. Containment refers to actions that staff take to limit the freedoms of patients in mental health care to mitigate or prevent conflict, including restraint, enforced sedation and seclusion (Bowers 2006; Doedens et al. 2020; Ngune et al. 2023).

**TABLE 1:** Conflict and containment items from Bowers (2006).

Conflict	Containment
Verbal aggression	Given as‐required medication
Physical aggression against objects	Given intramuscular injection (enforced)
Physical aggression against others	Transferred to psychiatric intensive care ward
Physical aggression against self	Seclusion
Suicide attempt	Intermittent observation
Smoking in a no smoking area	Continuous observation
Refusing to eat	Show of force
Refusing to drink	Physically restrained
Refusing to attend to personal hygiene	Time out
Refusing to get out of bed	Locking the ward door
Refusing to go to bed	Searching of bags and pockets
Refusing to see workers	Removing items
Alcohol misuse	Search bed space
Other substance misuse	Sniffer dogs
Absconding	No kitchen access
Refusing regular medication	No bathroom access
Refusing as‐required medication	Closed‐circuit television

Conflict and containment can lead to several negative outcomes for staff and patients (Bowers 2006). Patients subjected to high levels of containment, or who experience high levels of conflict, tend to have overall poor health outcomes (Doedens et al. 2020). Containment can re‐traumatise individuals (Sweeney et al. 2018) or lead to a perception of being abused, feelings of isolation/abandonment (Askew et al. 2019; Cutler et al. 2020, 2021) and iatrogenic mental health conditions such as post‐traumatic stress disorder (Chieze et al. 2019). Coerced‐containment in mental health care is thought to have an iatrogenic link to suicide (Large et al. 2014; Borecky et al. 2019; Jordan and McNiel 2020; Ward‐Ciesielski and Rizvi 2021). Containment is also detrimental to nurses, where staff report higher rates of physical and moral injury after engaging in restrictive care (Lancaster et al. 2008; Ye et al. 2019).

A systematic review by Ngune et al. (2023) aimed to identify nursing variables that could be associated with patient‐related (safety) outcomes in acute mental health care settings. Ngune et al. (2023) reported that aggression, seclusion, restraint use, self‐harm, absconding, PRN medications and special observations can be used as nursing‐sensitive indicators of the quality of care (Ngune et al. 2023). However, the association between the number of nursing staff, or variation in the composition of the nursing team, and incidents has not been fully established (Baker et al. 2019; Thibaut et al. 2019; Woodnutt 2023; Thompson et al. 2024), and this could be a modifiable factor that is associated with reductions in adverse incidents.

There is ample evidence that nurse staffing levels (e.g., the number of nurses per shift, per patient or nurse hours per patient day) and skill‐mix (a measure of the proportion of the nursing staff who are registered nurses, higher‐qualified or a measure of the years of experience of the nursing team) are associated with the safety and quality of care in general/somatic healthcare settings (Kane et al. 2007; Ball et al. 2014, 2018; Griffiths et al. 2018; Twigg et al. 2019; Ball and Griffiths 2022). These relationships have informed methods to estimate the nurse staffing required to provide safe care in general medical settings (Griffiths et al. 2020). While there is insufficient evidence to determine the best approach to identify optimal staffing levels (Griffiths et al. 2020), there is clear evidence that more nurses lead to fewer adverse incidents, missed‐care and mortality (McHugh et al. 2021; Willis and Brady 2022). Longitudinal analyses allow us to infer that inadequate staffing has a causal relationship with patient mortality (Dall'Ora et al. 2022). Several patient outcomes are sensitive to nurse staffing, notably missed nursing care and increased likelihood of errors. Consequences include compromised care: inadequate assessments and care omissions place patients at increased risk of complications, adverse events and death (Ball et al. 2018; Griffiths et al. 2018).

Health services currently employ and deploy mental health nurses in diverse manners and settings despite a lack of synthesised data and analyses concerning effectiveness (Phoenix 2019). United Kingdom (UK) guidance on safer staffing in mental health services is piecemeal, does not advise on the volume, specific skills or training of staff, but suggests staffing levels need to be proportionate to patient risk (NAPICU 2014; Royal College of Psychiatrists 2020). A UK framework for the composition of health professions that contribute to ward care (such as medicine, nursing, occupational therapy and social work) is available although this does not specify nursing volume and has not been updated recently (NHS England 2015). In New South Wales, Australia, a mental health nurse‐to‐patient ratio minimum (1:4–1:7, respectively, depending on shift) has been mandated by the Nurses and Midwives Association, although it is not clear on what evidence this has been based (NSW Nurses and Midwives Association 2022). Staffing levels or skill‐mix guidance specific to mental health settings needs to be underpinned by a full review of the available evidence. Such a review is currently lacking; therefore, the aim of this systematic review was to identify and synthesise evidence of the association between nurse staffing levels and skill‐mix and incidents in acute inpatient mental health settings.

## 2 Methods

### 2.1 Design

This was a systematic literature review and is reported according to the Preferred Reporting Items for Systematic Reviews and Meta‐Analyses (PRISMA) guidelines (Page et al. 2021). This review considered two staffing configurations: the number of nursing staff providing care for patients (staffing levels) and the skill‐mix within the nursing team. Staffing levels were defined as any measure of the number of nursing staff over the number of patients and/or beds in a unit: such as staff‐to‐patient ratio, staff‐to‐bed ratio, staff hours per patient day. Skill‐mix was defined in terms of the ratio between professional and non‐professional staff within the nursing team (e.g., proportion of registered nurses vs. registered nurses and assistant staff) or skill within the professional nursing team (e.g., measures of experiences or specialists qualifications).

Many of the conflict and containment items are not routinely reported (Ward‐Stockham et al. 2022; Woodnutt 2023), or do not appear in a detectable frequency in datasets or published research (Thibaut et al. 2019). Therefore, incidents that mirrored high‐frequency reporting in our previous analysis of English data were included (Woodnutt et al. 2024).

Conflict variables included were: verbal aggression, aggression against objects, aggression against others, aggression against self, suicide attempts and refusal of medication (regular and as‐required). Containment variables considered were: seclusion, restraint and the use of sedation.

### 2.2 Search Strategy

**FIGURE 1:**
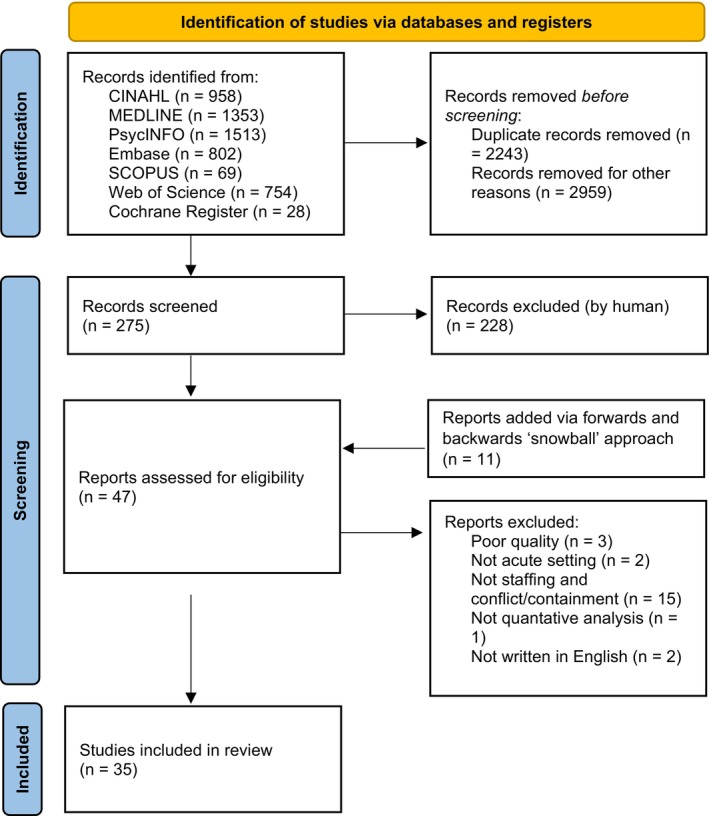
PRISMA 2020 flow diagram for new systematic reviews which included searches of databases and registers only. *Source:* Page et al. (2021). This work is licensed under CC BY 4.0. To view a copy of this licence, visit https://creativecommons.org/licenses/by/4.0/.

Our search comprised free‐text terms and subject headings related to nurse staffing levels, mental health settings and conflict and containment incidents (full terms are in Table S1). We searched CINAHL, Cochrane, Embase, MEDLINE, PsycINFO, SCOPUS and Web of Science in May 2024. Full details of the search terms can be found in Table S1. Following full‐text screening, a manual forward (included studies' cited by data) and backwards (reference lists of included studies) ‘snowball’ search was performed. Further details can be found in Figure 1. Full selection criteria are reported in Table 2. We included studies written in English and excluded studies that did not include an acute mental health care setting (e.g., solely forensic/secure mental health settings). We also excluded studies whose focus was multifactorial change in policy or procedure where the effect of staffing change could not be isolated. S.W. performed the searches and selection with supervision from P.G. and C.D.O.; discrepancies were resolved through discussion until consensus was reached.

**TABLE 2:** Eligibility criteria.

	Inclusion criteria	Exclusion criteria
Population	Adult patients (aged over 18 years) in acute mental health care or whole trust/hospital settings. No restrictions were imposed on ward or hospital size	All specialist mental health ward provision including forensic mental health. All child and adolescent and older person's mental health wards. General acute medical/surgical wards/hospitals
Intervention	Nurse staffing levels within a defined ward, hospital size (e.g., as a ratio of nurses to patients/beds) or a given time period (e.g., number of nurses per shift). Deviation in nurse‐staffing levels (e.g., % change in actual vs. rostered) Nursing skill‐mix: proportion of nurses who are registrants or the experience levels of staff (measure of time in role or position)	Evaluation of training packages, qualification level of nursing staff Studies examining nurse perception/psychological state Studies evaluating a service improvement where staffing levels or skill‐mixed were modified, but their relationship to incidents could not be separated from other measures in analyses
Outcome	Conflict: verbal aggression, aggression against objects, aggression against others aggression against self, suicide attempts and refusal of medication (regular and pro re nata). Containment: seclusion, restraint and the use of sedation Composite conflict and containment outcomes will be considered, provided most incidents reported fall within the above definitions	
Study design	Empirical (observational or experimental) studies of nurse staffing and conflict and/or containment	Qualitative studies, literature and systematic reviews, grey literature

### 2.3 Data Extraction, Synthesis and Quality Assessment

Data were extracted using a standardised form, covering staffing and safety variables, study design, participants, effect size, confidence intervals, significance (*p*‐value) and summary findings. Full details are in Table 2 and Table S4.

Due to significant variation in staffing measures and analysis methods, a statistical meta‐analysis was not possible; therefore, we adopted a narrative synthesis approach according to Campbell et al. (2020). Synthesis without meta‐analysis (SWiM) (Campbell et al. 2020) considers prioritisation of methodological quality for inclusion in synthesis. For this review, the priorities for reporting results were: quality assessment, sample size, design, degree of error in measurement of conflict and containment and staffing variables, statistical significance, magnitude of effect size (adapted from Cohen 1988; Chen et al. 2010), precision (confidence intervals) and generalisability. Effect size was recorded on a seven‐point scale from −3 (large inverse effect) to 3 (large positive effect); non‐significant (*p* > 0.05) or non‐reported results were given a magnitude of 0. Further details and effect sizes can be found in Table S3. We used R version 4.2.2 (R Core Team 2024) and the tidyverse (ver 2.0.0) package (Wickham et al. 2019) to produce plots.

Quality Assessment was conducted by S.W. using tools from the Joanna Briggs Institute that matched the methodology of included designs through a sum of the number of positive items (Porritt et al. 2014; Joanna Briggs Institute 2023). The appraisal tool score was converted to a percentage (where 100% = highest quality) for comparison between studies using different methods. S.W. conducted data extraction and quality assessment. Decisions and extractions were reviewed in meetings to resolve discrepancies, with notes and reports shared with authors P.G. and C.D.O. throughout the process.

## 3 Results

### 3.1 Search Results

A total of 5477 studies were identified, with 35 meeting the inclusion criteria. All were observational. Twenty‐four studies were cross‐sectional, six were cohort, and five were case–control studies. Sample sizes ranged from 24 to 70 136. Studies varied in scope, including two comparing multiple countries, two across hospitals in the same organisation, 14 with national data from multiple organisations, nine across multiple wards in one hospital and eight at a single ward level.

Staffing levels were described in five ways: (i) staff‐to‐bed ratio, (ii) staff‐to‐patient ratio, (iii) deviation from planned staffing, (iv) staffing levels over a specific time period (e.g., 24 h) and (v) staff‐completed ratings of ward staffing. Nursing skill‐mix was defined in two ways: (i) staff experience levels and (ii) the proportion of registered nurses.

Seven studies drew samples from data collected for the City‐128 study; a sample of 136 wards across 67 hospitals in the UK from 2004 to 2005 (Bowers et al. 2013). Three studies by Doedens et al. (2017, 2020, 2021) also contain overlapping data and were drawn from a ward in the Netherlands. Two studies by Staggs (2015, 2016) contained overlapping samples drawn from a national database of American health data. Many studies incorporated multiple eligible analyses (e.g., multiple staffing level, skill‐mix or conflict/containment measurements). In total, there were 58 reported analyses of the association of staffing levels or skill‐mix with conflict and containment.

All studies included data from acute psychiatric wards in high‐income countries. Ten studies were from the UK, nine from the USA, five from the Netherlands, one from 10 European countries and one comparing Norway and Denmark. Other studies were conducted in Australia, Canada, Denmark, Japan, New Zealand, Norway, South Korea, Sweden and Taiwan.

### 3.2 Quality Assessment

Most included studies were of low to moderate quality, and only five studies (Kalisova et al. 2014; Fukasawa et al. 2018; Park et al. 2020; Doedens et al. 2021, 2022) were rated positively against ≥ 80% of the criteria. Full study characteristics are available in Table S4.

Most studies had limitations in the lack of standardisation of measurement of the outcome (conflict and containment) variable. Most studies used routinely collected data derived from incident reports. Reliance on incident reports in most studies means that there is a pervasive risk of under‐reporting of the outcome, with potential systematic bias related to staffing. Three studies used direct observation by a researcher to gather data (Doedens et al. 2017, 2021, 2022) instead of relying on staff self‐reports or routine data.

Only six studies (Husum et al. 2010; Doedens et al. 2017, 2021, 2022; Fukasawa et al. 2018; Park et al. 2020) adjusted for patient variables (such as acuity of illness, diagnosis, etc.) using multivariable analysis. Husum et al. (2010) considered variables such as patient length of stay, ward location, diagnosis, gender, intoxication and clinical measures (e.g., Global Assessment of Functioning [GAF]). Fukasawa et al. (2018) examined demographic and clinical factors, including age, gender, diagnoses, legal status, medication dosage and GAF. Park et al. (2020) adjusted for age, gender, diagnoses, previous admissions and insomnia, but excluded variables like intoxication or medication use (Doedens et al. 2017, 2021, 2022) accounted for both patient and staffing factors, including staff characteristics such as physical stature. Other studies (e.g., Bak et al. 2015; Staggs 2015, 2016) adjusted only for hospital or ward‐related factors such as the size of wards (e.g., census), function of wards (e.g., long‐stay vs. short‐stay acute wards) or settings (e.g., rural vs. urban) with the remainder including no control for case mix.

Studies with higher quality (relative to other included studies) contained larger sample sizes yet relied on routinely collected data on conflict and containment. These studies demonstrated greater precision in their confidence intervals, spanned longer time frames, and used multivariable analyses to adjust for confounding, effect modifying or mediating factors. In contrast, studies with lower quality typically had smaller sample sizes and relied on repeated safety incident reports from a limited patient population, which may not represent broader trends. Furthermore, studies conducted during periods of policy changes aimed at reducing incidents often faced confounding factors, as changes in practice or regulations may have influenced outcomes, limiting the generalisability and validity of their findings. Further details of quality appraisal can be found in Tables S6–S8.

### 3.3 Conflict and Containment Outcomes

Table 3 summarises staffing and outcome measurements across all studies. Conflict was defined as aggression or violence in 14 studies (e.g., Chou et al. 2002; Staggs 2015; Doedens et al. 2022), as a composite variable (aggression all types, or all types of conflict) in three (Bowers 2009; Bowers and Crowder 2012; Bowers et al. 2013), as medication refusal or discretionary nurse use in one (Baker et al. 2009) and as self‐harm in one (Bowers et al. 2008). Containment was measured as seclusion (supervised confinement) in 11 studies (e.g., Bak et al. 2015; Fukasawa et al. 2018), restraint (manual or mechanical) in six (e.g., Bak et al. 2015; Kodal et al. 2018) and as a composite variable in five (e.g., Kalisova et al. 2014; Park et al. 2020).

**TABLE 3:** Staffing level and skill‐mix operationalisations, safety variables and significance.

Staffing measure (number of studies using this measure)	Patient safety measure (number of studies combining staffing and safety measure)	Author(s) (year)	Studies where staffing measure was significantly associated with adverse incident reporting	Overall effect
Staffing level				
Staff‐to‐patient ratio (13)	Seclusion	Betemps et al. (1993); Donat (2002); Janssen et al. (2007); Bak et al. (2015)^a^; Doedens et al. (2017, 2021, 2022)	Donat (2002)	+
	Aggression	Lanza et al. (1994, 1997); Staggs (2013, 2016)	Staggs (2013)	−
	Restraint	Donat (2002)		
	Aggression to patients	Staggs (2015)	Staggs (2015)	+
	Aggression to staff	Staggs (2015)	Staggs (2015)	−
	Containment (composite)	Park et al. (2020)	Park et al. (2020)	+
	Sedation	Park et al. (2020)	Park et al. (2020)	−
Staff‐to‐bed ratio (11)	Conflict and containment (composites)	Bowers (2009); Bowers and Crowder (2012); Bowers et al. (2013)	Bowers (2009); Bowers and Crowder (2012)	−
	Seclusion	Husum et al. (2010); Fukasawa et al. (2018)	Fukasawa et al. (2018)	−
	Restraint	Husum et al. (2010); Fukasawa et al. (2018)	Fukasawa et al. (2018)	−
	Self‐harm	Bowers et al. (2008)	Bowers et al. (2008)	+
	Medicine refusal	Baker et al. (2009)	Baker et al. (2009)	+
	Involuntary sedation	Husum et al. (2010)		
	Restraint	Bowers et al. (2012)		
	Containment (composite)	Kalisova et al. (2014)		
	Aggression	Palmstierna and Wistedt (1995); Bowers et al. (2009)	Bowers et al. (2009)	−
Deviation from planned staffing level (4)	Aggression	Owen et al. (1998)		
	Restraint	Kodal et al. (2018)		
	Conflict and containment (composite)	Cook et al. (2020)		
	Seclusion	De Cangas (1993)		
Total staffing hours per given time period (shift/day) (3)	Seclusion	Morrison and Lehane (1995); O'Malley et al. (2007)	O'Malley et al. (2007)	+
	Seclusion and restraint	Pollard et al. (2007)		
Staff rating/report of staffing levels and environment (1)	Aggression	Rogerson et al. (2021)	Rogerson et al. (2021)	+
Skill‐mix				
Experience level (9)	Seclusion	Janssen et al. (2007); O'Malley et al. (2007); Doedens et al. (2017, 2021)		
	Aggression	Owen et al. (1998); Chou et al. (2002); Weltens et al. (2023)	Chou et al. 2002 (+); Weltens et al. (2023) (−)	+/−
	Containment (composite)	Williams and Myers (2001)		
	Restraint	Bak et al. (2015)		
Proportion of registered nurses (3)	Containment (composite)	Williams and Myers (2001)	Williams and Myers (2001)	+
	Aggression	Staggs (2013); Doedens et al. (2022)	Staggs (2013)	+

*Note:* “+” Indicates statistically significant association in favour of higher staffing level or skill‐mix. “−” Indicates statistically significant association in favour of lower staffing level or skill‐mix.

^a^ Relates to one sub‐sample (study drew two samples from two different countries)—the other sub‐sample was not significant.

One study (Cook et al. 2020) included all reported incidents, noting that a significant proportion were aggression‐related.

### 3.4 Staffing Levels and Conflict and Containment

Staffing levels were analysed in 32 studies using various approaches, with many conducting multiple analyses on staffing and its relationship to conflict or containment. Thirteen studies focused on staff‐to‐patient ratios, 11 on staff‐to‐bed ratios, 4 on deviations from planned staffing levels and 3 on total staffing hours. (Rogerson et al. 2021) used a composite measure of staffing and space which included staff‐to‐patient ratios alongside other aspects of the facility.

**FIGURE 2:**
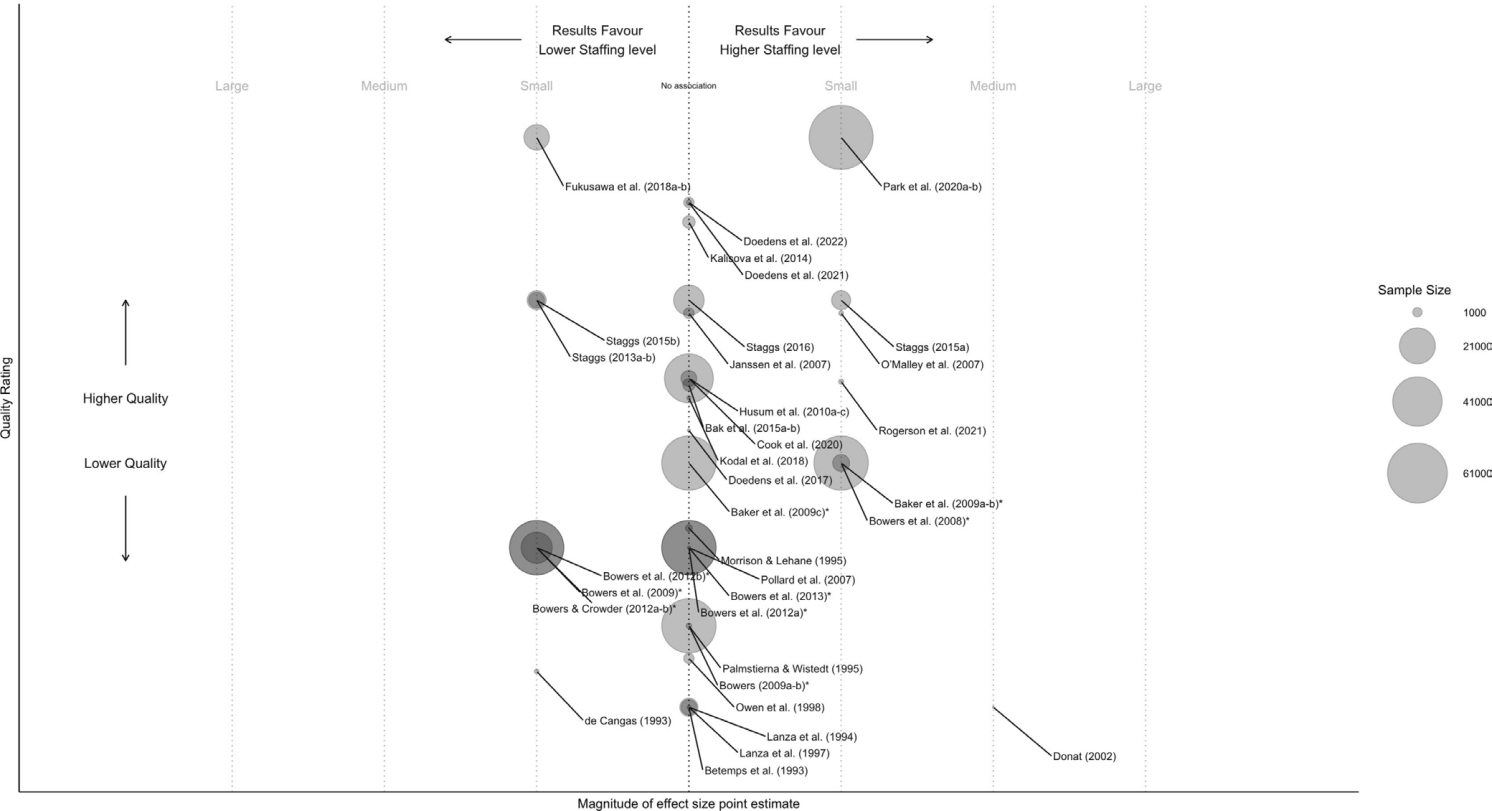
Nurse staffing level point estimate magnitude, study quality and sample size. *Studies drawn from same sample. a–c = multiple measures from same sample, direction or effect for non‐significant results is not specified.

Figure 2 summarises the findings, which were mixed. Some stronger studies (e.g., Staggs 2015; Park et al. 2020) found marginally reduced conflict and containment associated with increased staffing, while others (e.g., Bowers 2009; Fukasawa et al. 2018) reported small increases under similar conditions. Overall, among 44 analyses from 32 studies, equal numbers found increased (*n* = 9) and decreased (*n* = 9) conflict and containment when staffing levels rose. However, most analyses (*n* = 26) reported no association, with 18 not specifying parameters.

Three methodologically robust studies warrant further discussion. Fukasawa et al. (2018) in a study of 10 013 psychiatric admissions in Japan found that increased nursing staff per 10 patient beds doubled the likelihood of seclusion (aOR = 2.36, CI = 1.55–3.60) and increased restraint (aOR = 1.74, CI = 1.35–2.24). Conversely, Park et al. (2020) analysed 70 136 South Korean health insurance records and found that higher staff‐to‐patient ratios were linked to reduced psychiatric emergency treatments (aOR = 0.92, CI = 0.84–1.00, *p* < 0.05).

Staggs (2015) conducted a retrospective analysis of 2 years of U.S. psychiatric inpatient data, differentiating between aggression directed towards staff and patients. The study identified a paradoxical relationship: higher nursing hours per patient day were associated with an increase in assaults on staff (RR = 1.11, CI = 1.02–1.21, *p* = 0.015) but a decrease in assaults on other patients (RR = 0.81, CI = 0.71–0.93, *p* = 0.004).

### 3.5 Nursing Skill‐Mix and Conflict and Containment

**FIGURE 3:**
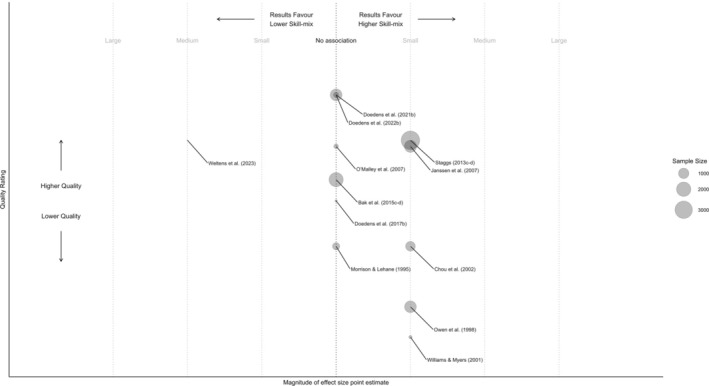
Nursing skill‐mix point estminate magnitude, study quality and sample size. a–b = multiple measures from same sample, direction or effect for non‐significant results is not specified.

Figure 3 summarises studies on skill‐mix and conflict/containment, plotting point estimates, sample sizes and quality. Comparatively, fewer studies examined the skill‐mix of staff where 12 studies reported 14 analyses: six found that higher skill‐mix was associated with reduced incidents, seven found no significant relationship and one found higher skill‐mix was associated with increased incidents.

Staff experience showed mixed associations with conflict and containment, with three analyses reporting significant links. Chou et al. (2002) found more nursing experience was associated with reduced aggression (OR = 0.91, CI = 0.84–0.98, *p* < 0.05), though a concurrent violence reduction program may have influenced results. Owen et al. (1998) reported increased aggression with fewer mental health nurses (RR = 1.35, CI = 1.09–1.68, *p* = 0.006). In contrast, Weltens et al. (2021) found greater experience was associated with more aggression reporting (OR = 3.5, CI = 1.32–8.26, *p* < 0.05), though the sample was small and the duration of the study short. Four studies (Morrison and Lehane 1995; Janssen et al. 2007; Bak et al. 2015; Doedens et al. 2017) reported non‐significant reductions in conflict and containment with increased experience, though Morrison and Lehane (1995) did not report parameters. Two studies (O'Malley et al. 2007; Doedens et al. 2021) found no association, but neither provided parameters.

### 3.6 Proportion of Registrants

Staggs (2013) found that a higher proportion of registered nurses was associated with reduced aggression and violence (RR = 0.94, CI = 0.90–0.97, *p* < 0.05), while (Williams and Myers 2001) found that a higher registered nurse proportion was associated with greater use of least‐restrictive interventions (*r* = 0.379, *p* < 0.05). Doedens et al. (2022) reported no association between teams comprising all registered nurses (as opposed to a mixture with unregistered care assistants) and aggressive behaviour.

## 4 Discussion

This review synthesises quantitative studies on mental health nurse staffing levels, skill‐mix and patient safety incidents. Studies examining staffing levels used varied approaches, including staff‐to‐patient ratios, staff‐to‐bed ratios, deviations from planned staffing and total staffing hours. Findings were mixed, with approximately equal numbers of analyses reporting increased and decreased conflict and containment associated with higher staffing levels, while most found no significant association. Notably, three robust studies (Fukasawa et al. 2018; Park et al. 2020; Staggs 2015) highlighted contrasting results, with higher staffing linked to both increased and decreased incidents depending on the context.

Studies examining skill‐mix were fewer, with similarly inconsistent findings. While some evidence suggests that a higher proportion of experienced or registered staff may reduce incidents of conflict and containment, other studies reported no significant relationship. The heterogeneity in study designs, definitions of staffing measures and outcome reporting likely contribute to these mixed findings, limiting firm conclusions about the role of staffing levels and skill‐mix in managing conflict and containment.

The literature is predominantly observational, relying heavily on routine or staff‐reported data. However, despite concerns of systematic observer or measurement bias in routine data, differing significant associations were identified in different samples, indicating at least a partial explanatory relationship between staffing and conflict and containment. However, said bias may have led to an over‐ or underestimation of effects, making it difficult to determine whether higher staffing levels or improved skill‐mix genuinely enhance care or merely influence reporting behaviour. The results across studies suggest there is a mixed and likely context‐dependent relationship between staffing and conflict and containment.

Notwithstanding the above, findings on staffing levels are inconsistent, with studies supporting higher staffing levels counterbalanced by equally rigorous studies showing the opposite (see Figure 2). While Staggs' (2015) analysis suggests staffing levels may have a differential impact on various incident types, most studies relied on composite outcomes (e.g., measures of ‘all aggression’). Evidence on skill‐mix, particularly the proportion of registered psychiatric nurses, is more consistent but limited, making it difficult to establish clear patterns.

The impact of staff experience levels is unclear, with few studies examining this and reporting mixed results. All studies of experience levels lacked robust methods to control for covariates and had relatively small samples, limiting their reliability. While higher skill‐mix overall appears linked to reduced conflict and containment, methodological weaknesses, such as lack of adjustment for key patient‐related factors reduce the strength and generalisability of these findings.

As Griffiths et al. (2016) highlight, there are several sources of bias that are common in observational studies linking staffing to safety outcomes. Staffing levels could influence incident reporting, with higher staffing leading to more incidents being recorded. Additionally, without proper risk adjustment, higher staffing might reflect higher‐risk environments rather than a direct impact on safety. Incident categorisation is also crucial; Staggs (2015) showed that increases in one type of aggression may coincide with decreases in another, complicating the interpretation of composite variables (used by most included studies) and potentially obscuring true relationships.

Most studies did not adjust for case‐mix or account for environmental factors that may influence psychiatric outcomes. While seclusion was the most frequently studied clear outcome (assessed in 12 studies where analyses could be separated and considered independently), aggression—often a precursor to seclusion and other coercive interventions—has generally received minimal attention (Al‐Maraira and Hayajneh 2019). Aggression was most frequently analysed by studies as a composite variable, thus meaning the relationship between staffing and types of aggression (e.g., aggression to staff or aggression to patients) is impossible to discern at this time. Research shows that aggression reporting is also influenced by ward design features (Ulrich et al. 2018; Rogerson et al. 2021), as well as staff characteristics, such as years of experience and staff's personal/professional histories (Schlup et al. 2021).

The work of Doedens et al. (2017, 2021) further highlights that nursing staff characteristics, including gender, personality traits and stature, may also influence conflict and containment rates. However, no other included studies controlled for these factors in their multivariable analyses. These findings indicate that staffing levels or skill‐mix could partially explain variation in aggression and seclusion rates, although, due to issues with quality, the overall picture is unclear.

Most statistically significant results were identified through cross‐sectional analysis. Even among the studies that did adjust for case‐mix, results remain inconsistent, suggesting that there may be unmeasured confounding and/or lack of control for effect modifiers or mediators. For example, Fukasawa et al. (2018) found that higher staffing levels were associated with greater use of seclusion and restraint, while Park et al. (2020) reported the opposite—that higher staffing levels were linked to less restraint and reduced use of ‘psychiatric emergency treatment’ (sedation). However, neither study considered compositional aspects of the nursing team (e.g., how many nurses were substantively employed, how many nursing staff were or were not registered with a professional body); both of which could have plausibly influenced results. Lack of management for effect modifiers and mediators, including different staffing types and proportions, is a gap in available literature, and further studies that include this may begin to alleviate the lack of clarity.

The disparity in reported associations between studies underscores the complexity of the relationship between staffing levels and incidents, suggesting that the effects of staffing may be context‐dependent and influenced by other, unexamined factors. The inconsistency of findings, even among studies with stronger designs and case‐mix adjustment, highlights the need for more refined methodologies and a deeper exploration of the underlying mechanisms at play.

Findings linking higher staffing to increased incident reporting could arise in several ways. More staff lead to more patient interactions, increasing the likelihood of conflict, and may make staff more inclined to initiate containment procedures (Bowers 2009; Bowers et al. 2009; Staggs 2015). Planned interventions may also be delayed until sufficient staff are available, skewing results. Wards with higher patient numbers may see more interactions, further distorting data (Weltens et al. 2021). Additionally, registered staff may report incidents more frequently due to their expertise (Hamed and Konstantinidis 2022), and senior staff may be more likely to notice and report incidents. A survey of UK mental health nurses found that a third felt staffing levels were insufficient, with nearly half reporting compromised care (Thompson et al. 2024), suggesting that lowering staffing levels is unlikely to improve care.

Research in somatic/physical settings has linked indicators like missed‐care to outcomes like mortality, where the impact is predictable and clear (Dall'Ora et al. 2022). In contrast, mental health indicators often conflict with health service targets (Woodnutt 2023; Woodnutt et al. 2024). For instance, increased containment may reduce hospital length‐of‐stay (Park et al. 2020), a key UK health policy target (NHS 2019) and patients with higher risks may be assigned to wards with better staffing (Fukasawa et al. 2018). These complexities make it difficult to use certain outcomes as reliable proxies for care quality.

### 4.1 Limitations

There are two main limitations of this review. First, data extraction and quality appraisal were undertaken by a single reviewer (S.W.). However, key decisions were checked and errors in extraction are unlikely to alter the conclusions. Second, the review was limited to published peer‐reviewed literature, but it seems unlikely that a large body of unpublished literature of higher quality exists that might change the overall conclusions.

### 4.2 Implications for Policy, Practice and Research

While the evidence offers few, if any, direct policy implications, safe staffing in mental health settings remains a critical issue. Large‐scale, longitudinal research with better risk adjustment is needed. The current lack of clarity reflects the evolving field of mental health workforce research, where outcomes often have divergent interpretations (Woodnutt 2023; Woodnutt et al. 2024). There is a need to focus on how data can drive mental health nursing effectiveness (Rice et al. 2019), presenting an opportunity to better define and measure mental ill‐health and improve care delivery. Increasing the volume, proportion and skill of mental health nurses is a global goal (WHO 2022), though using data to guide staffing decisions remains challenging.

## 5 Conclusion

The current staffing crisis in mental health nursing underscores the urgent need for robust evidence to guide policies and improve patient outcomes. However, existing research on the relationship between staffing, skill‐mix and conflict/containment outcomes is limited by inconsistent findings, poor study design and inadequate adjustment for confounders. A key challenge lies in identifying meaningful indicators from administrative data that accurately reflect the safety and quality of care. While there is no evidence to support reducing staffing levels or diluting the skill‐mix to improve outcomes, further large‐scale studies are needed. These studies must control for known confounders, mediators and effect modifiers to better understand the impact of staffing on outcomes and inform effective workforce planning.

## 6 Relevance to Clinical Practice

This review summarises the literature on staffing levels and skill‐mix of inpatient mental health nurses in acute settings, focusing on high‐frequency incidents. Given the mixed and inconclusive evidence, no clear recommendations for workforce planning or deployment can be made. However, it highlights the need to reconsider the nature and efficacy of routinely collected information, and how data should be defined and collated to better inform workforce planning and policy. Mental health settings need tailored reporting categories, as workforce factors influencing key outcomes (e.g., self‐injury) likely differ from those in other settings. This would give clinicians and researchers clearer insights into improving safety and care quality. While we understand many aspects of patient harm in mental health environments, we know far less about the organisational and workforce factors that drive or prevent it.

## Author Contributions

Samuel Woodnutt designed the study and acquired the data (including selection and screening), computed this in the R programme and plotted the tables/graphs. Simon Hall and Paula Libberton helped draft the rationale for the study and the current context within mental health nursing. Chiara Dall'Ora, Jane Ball and Peter Griffiths reviewed drafts of the manuscript and provided supervision and conceptual guidance alongside editing work. Peter Griffiths was consulted to review the final include list and resolve discrepancies.

## Disclosure

Authorship: All the authors have fully met criteria of authorship, having: (1) Engaged in the conception and design of the study, acquisition of data or analysis and interpretation of data. (2) Drafted the article/revised it critically for important intellectual content. (3) Given final approval of the version submitted.

## Ethics Statement

Ethics not applied for as this was a systematic review of conventionally published literature.

## Consent

Patients were not involved so no patient consent was required.

## Conflicts of Interest

The authors declare no conflicts of interest.

## Supporting information


Data S1.

## Data Availability

The authors have nothing to report.
