# Supplementary material for: The Association Between Nurse Staffing and Conflict and Containment in Acute Mental Health Care: A Systematic Review
Source: Int J Ment Health Nurs. 2025 Apr 7;34(2):e70039. doi: 10.1111/inm.70039 (PMC11976120; doi:10.1111/inm.70039)
Supplement: Supplementary file 1 — Data S1. [file INM-34-0-s001.pdf]

Table 1: Search terms:

D1 Staff Descriptor	D2 Safety Descriptor	D3 Setting Descriptor
**1 registered**	5 violen*	19 "psychiatr* ward*"
**2 "mental health"**	6 agressi*	20 "mental health ward*"
**3 psychiatr***	7 assault*	21 "psychiatric intensive care"
**4 nurs* (prox. 3)**	8 disrupt*	22 "psychiatr* unit"
	9 "self harm*"	23 "mental health unit"
	10 "self injur*"	24 "psychiatr* hospital"
	11 ligatur*	25 "mental health hospital"
	12 "self mutilat*"	26 "mental health inpatient*"
	13 suicid*	27 "psychiatr* inpatient*"
	14 "medication refus*"	
	15 seclusion	
	16 restrain*	
	17 "rapid tranquil*"	
	18 sedat*	

((1 OR 2 OR 3) NEAR 4) AND (5 OR 6 OR 7 OR 8 OR 9 OR 10 OR 11 OR 12 OR 13 OR 14 OR 15 OR 16 OR 17 OR 18) AND (19 OR 20 OR 21 OR 22 OR 23 OR 24 OR 25 OR 26 OR 27)

Table 2: Search terms in EBSCOhost format:

(registered OR “mental health” OR psychiatr*) N3 nurs* AND (violen* OR agressi* OR assault* OR disrupt* OR “self harm” OR “self injur*” OR “self mutilat” OR “ligatur*” OR suicid* OR “medication refus*” OR seclusion OR restrain* OR “rapid tranquil*” OR sedat*) AND (“psychiatr* ward*” OR “mental health ward*” OR “psychiatric intensive care” OR “pschiatr* unit” OR “mental health unit*” OR “psychiatr* hospital*” OR “mental health hospital*” OR “mental health inpatient*” OR “psychiatr* inpatient*”)

Table 3: Magnitude of point estimate effect size boundaries used in this review:

Effect size index	Weak	Medium	Strong
Cohen’s d (reference)	0.2-0.49	0.5-0.79	≥0.8
Pearson’s r (inverted values used for inverse association)	0.10-0.39	0.40-0.69	=>0.70
Odds ratio/rate ratio (positive association)*	1.68-3.46	3.47-6.70	≥6.71
Odds ratio/rate ratio (inverse association)**	0.80-0.99	0.58-0.79	≤0.58

**from Chen, Cohen and Chen (2010)*
***adapted from Chen, Cohen and Chen (2010); values calculated through log transformation then exponentiated*

Table 4: Staffing Levels – included papers characteristics/data abstraction

Authors	Year	Safety variable	Staffing Variables	Study design & participants	Findings	Direction of effect
**Betemps, Somoza & Buncher**	1993	*Seclusion*	*Patient-to-staff ratio (average daily census divided by FTE staff).*	*Cross-sectional*<br><br>*n = 4796 seclusion incidents*	*No significant association: parameters not reported*	N
**De Cangas**	1993	*Seclusion*	*Percentage of full complement (actual staffing divided by demand).*	*Cohort*<br><br>*Shift-by-shift seclusion rates for 1 month +*<br>*n = 34 staff surveys*	*Reported a significant correlation between an increase in percentage of full complement and reduction in seclusion hours ($p < 0.05$) but no parameters reported.*	+
**Lanza et al.**	1994	*Aggression*	*Patient-staff ratio was divided into quartiles and compared with assault rate for each quartile.*	*Cohort*<br><br>*n = 74 assaults*	*Correlation parameters reported, no significant association.*	N
**Morrison & Lehane**	1995	*Seclusion*	*Staffing levels (all staff, staff-nurses and charge nurse) on shifts when seclusion was used, compared with levels when not used.*	*Case control*<br><br>*n = 450 shifts, 225 shifts with seclusion, 225 shifts without*	*No significant association: parameters not reported.*	N
**Palmstierna & Wistedt**	1995	*Aggression*	*Staffing levels remained the same whilst ward beds reduced from 19 to 10.*	*Case control*<br><br>*n = 241 aggression incidents*	*No significant association: parameters not analysed/reported*	N

Key:
* same dataset from City-128 study
^ overlapping datasets
~ overlapping datasets
FTE = Full-time equivalent staff
OR = Odds Ratio
aOR = adjusted odds ratio from multilevel analysis
r = Pearson's r
CI = 95% confidence interval
F = ANOVA F statistic, df = 3 and 132

Effect of nurse staffing on safety variable:

+	increase in staffing level variable increased likelihood of patient safety (less adverse events)
-	increase in staffing level variable decreased likelihood of patient safety (more adverse events)
N	no association between staffing and safety variable
+/-	Increase in staffing level increased safety in one variable, but decreased safety in other(s)

Table 4: Staffing Levels – included papers characteristics/data abstraction

**Lanza et al.**	1997	*Aggression*	*Mean volume of nurses on duty on shifts with an assault.*	*Cross-sectional*<br><br>*n = 3312 consecutive shifts (1543 assaults)*	*No association: parameters not reported.*	N
**Owen et al.**	1998	*Aggression*	*Number of required and actual staff.*	*Cohort*<br><br>*n = 1289 aggression incidents across 5 wards*	*No association: parameters not reported.*	N
**Donat**	2002	*Seclusion & restraint*	*Monthly average staffing level was divided by average daily patient census.*	*Cross-sectional*<br><br>*n = 2-year sample of 1 hospital data (24 months)*	*Significant association between an increase in staff-to-patient ratio and seclusion hours (r = -0.51, p<0.05).*	+
**Janssen et al.**	2007	*Seclusion*	*Patient census divided by the number of staff on shift.*	*Cohort*<br><br>*n = 1373 ward days across 10 wards*	*No significant association: parameters not reported.*	N
**O'Malley et al.**	2007	*Seclusion*	*Total nurse hours per shift.*	*Cohort*<br><br>*n = 846 seclusion incidents*	*Statistically significant association between an increase in the number of nursing staff (nurse hours) and decrease in seclusion rates (r = -0.25; p = 0.001) in univariate analysis. Multivariate analysis suggests that the period of study, shift and nurse hours explained 23% of the variance in seclusion use.*	+
**Pollard et al.**	2007	*Seclusion & restraint*	*Staffing Levels per 24 hours.*	*Cross-sectional*<br><br>*n = 46 months' collection in hospital*	*No significant association: parameters not reported.*	N

Key:
* same dataset from City-128 study
^ overlapping datasets
~ overlapping datasets
FTE = Full-time equivalent staff
OR = Odds Ratio
aOR = adjusted odds ratio from multilevel analysis
r = Pearson's r
CI = 95% confidence interval
F = ANOVA F statistic, df = 3 and 132

Effect of nurse staffing on safety variable:

+	increase in staffing level variable increased likelihood of patient safety (less adverse events)
-	increase in staffing level variable decreased likelihood of patient safety (more adverse events)
N	no association between staffing and safety variable
+/-	Increase in staffing level increased safety in one variable, but decreased safety in other(s)

Table 4: Staffing Levels – included papers characteristics/data abstraction

**Bowers et al.***	2008	*Self-harm*	*FTE nursing staff in post per bed.*	*Cross-sectional*<br><br>*>50,000 checklists from 136 wards*	*Greater presence of registered nurses at organisational level marginally reduced self-harm (OR = 0.94, CI = 0.90-0.98, p < 0.05). Parameters for ward and shift levels not reported.*	+
**Baker et al.***	2009	*Medicine Safety (containment)*	*FTE nursing staff in post per bed.*	*Cross-sectional*<br><br>*>50,000 checklists from 136 wards*	*Increased qualified nurse staffing was associated with regular medication refusal (OR = 0.94, CI = 0.94-0.98, p < 0.05), and demand for as-required medication (OR = 0.90, CI = 0.90 – 0.91, p < 0.05) at trust level. No parameters reported for ward or shift levels, or for refusal of as-required medication.*	+
**Bowers***	2009	*Conflict and containment (composites)*	*FTE nursing staff in post per bed.*	*Cross-sectional*<br><br>*>50,000 nursing checklists from 136 wards*	*An increase in the number of nurses per bed significantly increased containment (e.g. one unit increase in staffing numbers per bed per day increased conflict by one event every three days approximately) (coeff = 0.39, CI = 0.09-0.68, p < 0.05). No significant association: parameters not reported for containment.*	-
**Bowers et al.***	2009	*Aggression*	*FTE nursing staff in post per bed.*	*Cross-sectional*<br><br>*>50,000 checklists from 136 wards*	*An increase in qualified nurse staffing increased aggression to others (OR = 1.15, CI = 1.11 – 1.19, p < 0.05), aggression to objects (OR = 1.12, CI = 1.01 – 1.20, p < 0.05) and verbal aggression (OR = 1.03, CI = 1.02 – 1.04, p < 0.05) at ward and shift levels. Parameters for organisation level not reported.*	-
**Husum et al.**	2010	*Seclusion, restraint and involuntary sedation (containment)*	*Staff to bed ratio*	*Cross-sectional*<br><br>*3426 patient records from 32 acute wards*	*No significant association: parameters not reported.*	N

Key:
* same dataset from City-128 study
^ overlapping datasets
~ overlapping datasets
FTE = Full-time equivalent staff
OR = Odds Ratio
aOR = adjusted odds ratio from multilevel analysis
r = Pearson's r
CI = 95% confidence interval
F = ANOVA F statistic, df = 3 and 132

Effect of nurse staffing on safety variable:

+	increase in staffing level variable increased likelihood of patient safety (less adverse events)
-	increase in staffing level variable decreased likelihood of patient safety (more adverse events)
N	no association between staffing and safety variable
+/-	Increase in staffing level increased safety in one variable, but decreased safety in other(s)

Table 4: Staffing Levels – included papers characteristics/data abstraction

**Bowers & Crowder***	2012	*Conflict and containment (composites)*	*FTE nursing staff in post per bed.*	*Cross-sectional*<br><br>*15449 checklists completed as sub-sample: Nine shifts preceding rises in total conflict and containment*	*Time-series analysis revealed that rises in qualified nurse staffing preceded rises in total conflict and containment rates (multiple parameters reported, for both outcomes, strongest OR = 1.05, p < 0.05).*	-
**Bowers et al.***	2012	*Restraint and 'show of force'*	*FTE nursing staff in post per bed.*	*Cross-sectional*<br><br>*>50,000 nursing checklists from 136 wards*	*The likelihood of restraint (IRR = 1.21, CI 1.07-1.17, p<0.001) and 'Show of force' (IRR = 1.01, CI = 1.05-1.13, p<0.001) was not associated with the number of qualified nursing staff relative to the bed volume of the ward.*<br><br>*No association: parameters not reported for other staffing variables.*	N
**Bowers et al.***	2013	*Conflict and containment (composites)*	*FTE nursing staff in post per bed.*	*Cross-sectional*<br><br>*>50,000 checklists from 136 wards*	*Correlation study examining (sub-sample) wards with high conflict or low conflict and either high or low containment rates. The staffing level was higher in high conflict, high containment wards, no other noted associations: parameters not reported.*	N
**Staggs**	2013	*Aggression*	*Total nursing hours per patient day*	*Cross-sectional*<br><br>*n = 3397 ward months. Linear and spline models used for analysis.*	*No association between nursing hours per patient day and total assaults (OR = 1.12, CI = 1.08 – 1.16, p < 0.05) or injury assaults (OR = 1.12, CI = 1.06 – 1.18, p < 0.05).*	N
**Kalisova et al.**	2014	*Containment*	*Staff:patient ratio (per bed, per week)*	*Cross-sectional*<br><br>*n = 2027 patient records from 10 European countries*	*No significant association: parameters not reported.*	N

Key:
* same dataset from City-128 study
^ overlapping datasets
~ overlapping datasets
FTE = Full-time equivalent staff
OR = Odds Ratio
aOR = adjusted odds ratio from multilevel analysis
r = Pearson's r
CI = 95% confidence interval
F = ANOVA F statistic, df = 3 and 132

Effect of nurse staffing on safety variable:

+	increase in staffing level variable increased likelihood of patient safety (less adverse events)
-	increase in staffing level variable decreased likelihood of patient safety (more adverse events)
N	no association between staffing and safety variable
+/-	Increase in staffing level increased safety in one variable, but decreased safety in other(s)

Table 4: Staffing Levels – included papers characteristics/data abstraction

**Bak et al.**	2015	*Restraint*	*Patient-to-staff ratio*	*Cross-sectional*<br><br>*n = 183 hospitals (all psychiatric hospitals in Denmark and Norway)*	*No association for Patient – staff ratio (> 3 staff per patient vs. less) in Denmark (OR = 0.96, CI = 0.45-2.06, p > 0.05) or Norway (OR = 0.62, CI = 0.32-1.19, p = 0.15).*	N
**Staggs^**	2015	*Aggression*	*Registered nursing hours per patient day*	*Cross-sectional*<br><br>*461 wards over 2 years (minimum 6 months' data).*	*Higher staffing levels were associated with higher rates of assault against staff (OR = 1.11, CI = 1.02-1.21, p < 0.05) but lower rates of assault against patients (OR = 0.81, CI = 0.71 – 0.93, p < 0.05). No association for overall rate of assault.*	+/-
**Staggs^**	2016	*Aggression*	*Registered nursing hours per patient day*	*Cross-sectional*<br><br>*480 wards over 2 years as monthly data.*	*Fluctuations in staffing levels relative to the ward avereage was not assoiated with the odds of an assault occuring (parameters reported: no association).*	N
**Doedens et al.~**	2017	*Seclusion*	*Number of staff on shift when incident occurred*	*Cohort*<br><br>*23 incidents*<br>*47 staff on one ward.*	*No association: parameters not reported.*	N
**Fukusawa et al.**	2018	*Seclusion and restraint*	*Number of nurses per 10 beds (as ratio)*	*Cross-sectional*<br><br>*10013 patient admissions (total) across 113 wards.*	*Number of nurses per 10 beds was significantly associated with an increase use of seclusion (aOR = 2.36, CI = 1.55-3.60, p < 0.05) and restraint (aOR = 1.74, CI = 1.35 – 2.24, p < 0.05).*	-
**Kodal et al.**	2018	*Restraint*	*Total number of staff on duty & number of registered nurses per shift.*	*Case control*<br><br>*114 incidents of restraint in one ward*	*Total number of staff and number of registered nurses was not associated with the odds of restraint occurrence (parameters reported).*	N

Key:
* same dataset from City-128 study
^ overlapping datasets
~ overlapping datasets
FTE = Full-time equivalent staff
OR = Odds Ratio
aOR = adjusted odds ratio from multilevel analysis
r = Pearson's r
CI = 95% confidence interval
F = ANOVA F statistic, df = 3 and 132

Effect of nurse staffing on safety variable:

+	increase in staffing level variable increased likelihood of patient safety (less adverse events)
-	increase in staffing level variable decreased likelihood of patient safety (more adverse events)
N	no association between staffing and safety variable
+/-	Increase in staffing level increased safety in one variable, but decreased safety in other(s)

Table 4: Staffing Levels – included papers characteristics/data abstraction

**Cook et al.**	2020	*All adverse incidents*	*Variance between the number of clinically required and actual staffing.*	*Cross-sectional*<br><br>*n = 51 wards across 10 locations*	*No significant association for overall or registered nurse staffing and adverse incidents (parameters not reported).*	N
**Park et al.**	2020	*Containment*	*Staff to patient ratio (calculated as average monthly patient (including an 'adjusted' inpatient rate which incorporated day and out-patients) census divided by average monthly staffing level)*	*Cross-sectional*<br><br>*n = 70136 patient records from 453 hospitals (18379 patients in mental health wards)*	*Number of registered nurses to adjusted inpatient ratio decreased the likelihood of containment (OR = 0.96, CI = 0.92 – 1.00, p < 0.05). Total staffing to adjusted inpatient ratio also decreased the likelihood of containment (OR = 0.92, CI = 0.84 – 1.00, p < 0.05) and, in ordinal regression, increased sedation use by 1% (p < 0.05).*	+/-
**Doedens et al.~**	2021	*Seclusion*	*Number of staff on shift when incident occurred.*	*Cohort (prospective follow-up from 2017 study)*<br>*n = 112 seclusion incidents*	*No association: parameters not reported.*	N
**Rogerson et al.**	2022	*Aggression*	*Staffing levels defined within a rating tool completed by staff under 'environment'.*	*Cross-sectional*<br><br>*n = 191 staff surveys*	*Incident rate ratio for staffing levels when rated as higher by staff, compared to incidents of aggression being reported.*<br>*IRR 2.19, p < 0.05*	+

Key:
* same dataset from City-128 study
^ overlapping datasets
~ overlapping datasets
FTE = Full-time equivalent staff
OR = Odds Ratio
aOR = adjusted odds ratio from multilevel analysis
r = Pearson's r
CI = 95% confidence interval
F = ANOVA F statistic, df = 3 and 132

Effect of nurse staffing on safety variable:

+	increase in staffing level variable increased likelihood of patient safety (less adverse events)
-	increase in staffing level variable decreased likelihood of patient safety (more adverse events)
N	no association between staffing and safety variable
+/-	Increase in staffing level increased safety in one variable, but decreased safety in other(s)

Table 5: Skill-mix - included papers' characteristics/data abstraction

Authors	Year	Safety variable	Skill-mix variable	Study design & participants	Findings	Direction of effect
**Owen et al.**	1998	*Aggression*	*Experience level*	*Cohort*<br><br>*n = 1289 aggression incidents*	*No significant association: parameters not reported.*	N
**Williams & Myers**	2001	*Containment (composite)*	*Proportion of licensed nurses (of shift) and Experience level*	*Case control*<br><br>*n = 82 incidents requiring intervention*<br><br>*Study measured use of Least Restrictive Interventions (LRI) as an alternative to seclusion or restraint.*	*A greater proportion of registered nurses significantly reduced restraint use and increased least-restrictive intervention ($r = 0.379$, $p < 0.05$, one-tailed).*<br><br>*No significant associations with average years of experience and least restrictive intervention ($r = 0.146$, $p > 0.05$, one- tailed)*	+
**Chou et al.**	2002	*Aggression*	*Experience level*	*Case control*<br><br>*n = 855 incidents*	*Training received increased assault reporting (OR = 7.73, CI = 1.16-9.68, $p < 0.05$).*<br>*Staff with greater experience significantly reduced assault (OR = 0.91, CI = 0.84-0.98, $p < 0.05$).*	Training –<br><br>Experience +
**Janssen et al.**	2007	*Seclusion*	*Experience level*	*Cohort*<br><br>*n = 1373 ward days*	*Variability of work experience in nursing shift composition was significantly associated with a reduction in the use of seclusion. (OR = 0.871, CI = 0.828 – 0.938, $p < 0.05$). No other significant associations for other variables: parameters not reported.*	+
**O'Malley et al.**	2007	*Seclusion*	*Experience level*	*Cohort*<br><br>*n = 846 seclusion incidents*	*No significant association: parameters not reported.*	N

Key:
~ overlapping datasets
OR = Odds Ratio
r = Pearson's r
CI = 95% confidence interval

Effect of nurse staffing on safety variable:

+	increase in skill-mix variable increased likelihood of patient safety (less adverse events)
-	increase in skill-mix variable decreased likelihood of patient safety (more adverse events)
N	no association between staffing and safety variable

Table 5: Skill-mix - included papers' characteristics/data abstraction

**Staggs**	2013	*Aggression*	*Proportion of registered nursing hours in a monthly period*	*Cross-sectional*<br><br>*n = 3397 ward months. Linear and spline models used for analysis.*	*5% increase in the proportion of registered nursing hours reduced total assaults (OR = 0.94, CI = 0.90-0.98, p < 0.05) and injury assaults (OR = 0.94, CI = 0.90-0.98, p < 0.05) by 6% in the linear model.*<br><br>*Results from spline model were reported but not significant.*	+
**Bak et al.**	2015	*Restraint*	*Experience level*	*Cross-sectional*<br><br>*n = 183 hospitals (all psychiatric hospitals in Denmark and Norway)*	*Presence of staff with lower education levels decreased the frequency of restraint by 66% in Denmark (OR = 0.34, CI = 0.17-0.66, p < 0.01). All other parameters reported but not significant.*	+
**Doedens et al.~**	2017	*Seclusion*	*Experience level*	*Cohort*<br><br>*23 incidents*<br>*47 staff*	*Parameters reported but no significant association for all measures of skill-mix.*	N
**Doedens et al.~**	2021	*Seclusion*	*Experience level*	*Cohort (prospective follow-up from 2017 study)*<br><br>*n = 112 seclusion incidents*	*No significant associations: parameters not reported.*	N
**Doedens et al.~**	2022	*Aggression*	*Experience level*	*Cohort (prospective follow up including 2017 study data)*<br><br>*n = 1299 shifts*	*No significant association: parameters not reported.*	N
**Weltens et al.**	2023	*Aggression*	*Experience level*	*Cohort*<br><br>*n = 29 nurses who responded to bleep messages over 7 day period*	*More experienced nurses were associated with reporting more aggression (OR = 3.5, p < 0.05)*	+

Key:
~ overlapping datasets
OR = Odds Ratio
r = Pearson's r
CI = 95% confidence interval

Effect of nurse staffing on safety variable:

+	increase in skill-mix variable increased likelihood of patient safety (less adverse events)
-	increase in skill-mix variable decreased likelihood of patient safety (more adverse events)
N	no association between staffing and safety variable

Table 6: JBI Tool scoring for cross-sectional designs

Year	Title	Author	1. Were the criteria for inclusion in the sample clearly defined?	2. Were the study subjects and the setting described in detail?	3. Was the exposure measured in a valid and reliable way?	4. Were objective, standard criteria used for measurement of the condition?	5. Were confounding factors identified?	6. Were strategies to deal with confounding factors stated?	7. Were the outcomes measured in a valid and reliable way?	8. Was appropriate statistical analysis used?	Score (out of 8)	Score as %
1993	Hospital characteristics, diagnoses, and staff reasons associated with use of seclusion and restraint	Betemps et al.	**Y**	**N**	**U**	**U**	**N**	**N**	**N**	**U**	1	12.5
1994	Environmental Characteristics Related to Patient Assault	Lanza et al.	**Y**	**U**	**U**	**U**	**N**	**N**	**U**	**N**	1	12.5
1995	Changes in the pattern of aggressive behaviour among inpatients with changed ward organization	Palmstierna & Wistedt	**Y**	**Y**	**N**	**N**	**N**	**N**	**U**	**N**	2	25
1997	Original Articles Staffing of Inpatient Psychiatrsic Units and Assault by Patients	Lanza et al.	**Y**	**U**	**U**	**U**	**N**	**N**	**U**	**U**	1	12.5
2002	Impact of improved staffing on seclusion/restraint reliance in a public psychiatric hospital	Donat	**U**	**Y**	**N**	**U**	**U**	**U**	**N**	**N**	1	12.5

2007	Organizational and Unit Factors Contributing to Reduction in the Use of Seclusion and Restraint Procedures on an Acute Psychiatric Inpatient Unit	Pollard et al.	**Y**	**Y**	**Y**	**U**	**N**	**U**	**U**	**N**	3	37.5
2008	Relationship between service ecology, special observation and self-harm during acute in-patient care: City-128 study*	Bowers et al	**Y**	**Y**	**U**	**Y**	**N**	**N**	**Y**	**N**	4	50
2009	Wards features associated with high rates of medication refusal by patients: a large multi-centred survey*	Baker et al.	**Y**	**Y**	**U**	**Y**	**N**	**N**	**Y**	**N**	4	50
2009	Association between staff factors and levels of conflict and containment on acute psychiatric wards in England*	Bowers	**Y**	**Y**	**U**	**U**	**U**	**N**	**U**	**N**	2	25
2009	Identifying key factors associated with aggression on acute inpatient psychiatric wards*	Bowers et al.	**Y**	**Y**	**U**	**U**	**U**	**N**	**Y**	**N**	3	37.5
2010	A cross-sectional prospective study of seclusion, restraint and involuntary medication in acute psychiatric wards: patient, staff and ward characteristics	Husum et al.	**Y**	**Y**	**Y**	**Y**	**U**	**U**	**Y**	**U**	5	62.5
2012	Nursing staff numbers and their relationship to conflict and containment rates on psychiatric wards-A cross sectional time series Poisson regression study*	Bowers & Crowder	**Y**	**Y**	**Y**	**U**	**U**	**N**	**U**	**N**	3	37.5
2012	Manual restraint and shows of force: the City-128 study*	Bowers et al.	**Y**	**Y**	**Y**	**U**	**U**	**N**	**U**	**U**	3	37.5
2013	Correlation between levels of conflict and containment on acute psychiatric wards: the city-128 study*	Bowers et al.	**Y**	**Y**	**Y**	**U**	**U**	**N**	**U**	**N**	3	37.5
2013	Nurse Staffing, RN Mix, and Assault Rates on Psychiatric Units	Staggs	**Y**	**Y**	**Y**	**Y**	**U**	**N**	**Y**	**Y**	6	75
2014	Do patient and ward-related characteristics influence the use of coercive measures? Results from the EUNOMIA international study	Kalisova	**Y**	**Y**	**Y**	**Y**	**Y**	**Y**	**U**	**Y**	7	87.5
2015	Comparing the effect of non-medical mechanical restraint preventive factors between psychiatric units in Denmark and Norway	Bak et al.	**Y**	**Y**	**Y**	**Y**	**U**	**N**	**Y**	**U**	5	62.5
2015	Injurious Assault Rates on Inpatient Psychiatric Units: Associations With Staffing by Registered Nurses and Other Nursing Personnel ^	Staggs	**Y**	**Y**	**Y**	**Y**	**U**	**N**	**Y**	**Y**	**6**	75
2016	Deviations in monthly staffing and injurious assaults against staff and patients on psychiatric units ^	Staggs	**Y**	**Y**	**Y**	**Y**	**U**	**N**	**Y**	**Y**	6	75
2018	Relationship between the use of seclusion and mechanical restraint and the nurse-bed ratio in psychiatric wards in Japan	Fukusawa et al.	**Y**	**Y**	**Y**	**Y**	**Y**	**Y**	**Y**	**Y**	8	100

2020	An observational study on the rate of reporting of adverse event on healthcare staff in a mental health setting: An application of Poisson expectation maximisation analysis on nurse staffing data	Cook et al.	**Y**	**Y**	**Y**	**U**	**U**	**U**	**Y**	**Y**	5	62.5
2020	Nurse Staffing and Health Outcomes of Psychiatric Inpatients: A Secondary Analysis of National Health Insurance Claims Data	Park et al.	**Y**	**Y**	**Y**	**Y**	**Y**	**Y**	**Y**	**Y**	8	100
2022	The relationship between inpatient mental health ward design and aggression	Rogerson et al.	**Y**	**Y**	**Y**	**U**	**U**	**N**	**Y**	**Y**	5	62.5
2023	Staff and ward factors associated with aggression development on an acute closed psychiatric ward: an experience sampling method study	Weltens et al.	**Y**	**Y**	**Y**	**Y**	**U**	**N**	**Y**	**Y**	6	75

Table 7: JBI tool scoring for case-control designs:

**Year**	**Title**	**Author**	1. Were the groups comparable other than the presence of disease in cases or the absence of disease in control?	2. Were cases and controls matched appropriately?	3. Were the same criteria used for identification of cases and controls?	4. Was exposure measured in a standard, valid and reliable way?	5. Was exposure measured in the same way for cases and controls?	6. Were confounding factors identified?	7. Were strategies to deal with confounding factors stated?	8. Were outcomes assessed in a standard, valid and reliable way for cases and controls?	9. Was the exposure period of interest long enough to be meaningful?	10. Was appropriate statistical analysis used?	**Score (out of 10)**	**Score as %**

1995	Staffing levels and seclusion use	Morrison & Lehane	**U**	**U**	**Y**	**Y**	**Y**	**U**	**N**	**Y**	**U**	**N**	4	40
1998	Violence and aggression in psychiatric units	Owen et al.	**U**	**U**	**U**	**U**	**U**	**U**	**U**	**Y**	**Y**	**N**	2	20
2001	Relationship of Less Restrictive Interventions with Seclusion/Restraints Usage, Average Years of Psychiatric Experience, and Staff Mix	Williams & Myers	**U**	**U**	**U**	**U**	**Y**	**N**	**U**	**U**	**N**	**N**	1	10
2002	Factors Relevant to Patient Assaultive Behavior and Assault in Acute Inpatient Psychiatric Units in Taiwan	Chou et al	**U**	**U**	**U**	**Y**	**Y**	**N**	**N**	**Y**	**Y**	**N**	4	40
2018	Mechanical restraint and characteristics of patient, staff and shifts in a psychiatric ward	Kodal et al.	**Y**	**U**	**Y**	**Y**	**Y**	**N**	**N**	**Y**	**Y**	**N**	6	60

Table 8: JBI tool scoring for cohort studies

Year	Title	Author	1. Were the two groups similar and recruited from the same population?	2. Were the exposures measured similarly to assign people to both exposed and unexposed groups?	3. Was the exposure measured in a valid and reliable way?	4. Were confounding factors identified?	5. Were strategies to deal with confounding factors stated?	6. Were the groups/participants free of the outcome at the start of the study (or at the moment of exposure)?	7. Were the outcomes measured in a valid and reliable way?	8. Was the follow up time reported and sufficient to be long enough for outcomes to occur?	9. Was the follow up complete, and if not, were the reasons to loss to follow up described and explored?	10. Were strategies to address incomplete follow up utilized?	11. Was appropraite statistical analysis used?	**Score (out of 11)**	Score as %
1993	Nursing staff and unit characteristics: do they affect the use of seclusion?	de Cangas	**U**	**U**	**U**	**N**	**U**	**Y**	**Y**	**N**	**N**	**N**	**N**	**2**	18.181818

2007	The influence of staffing levels on the use of seclusion	Janssen et al.	**Y**	**Y**	**Y**	**N**	**N**	**Y**	**Y**	**Y**	**Y**	**Y**	**U**	**8**	72.727273
2007	Factors influencing seclusion rates in an adult psychiatric intensive care unit	O'Malley et al.	**Y**	**Y**	**Y**	**N**	**N**	**Y**	**Y**	**Y**	**Y**	**Y**	**U**	**8**	72.727273
2017	Nursing staff factors contributing to seclusion in acute mental health care—An explorative cohort study~	Doedens et al.	**Y**	**Y**	**Y**	**U**	**U**	**Y**	**Y**	**U**	**Y**	**U**	**N**	**6**	54.545455
2021	Influence of nursing staff characteristics on seclusion in acute mental health care—A prospective two-year follow-up study~	Doedens et al.	**Y**	**Y**	**Y**	**Y**	**Y**	**Y**	**Y**	**Y**	**Y**	**U**	**Y**	**10**	90.909091
2022	Association between characteristics of nursing teams and patients' aggressive behavior in closed psychiatric wards	Doedens et al.	Y	Y	Y	Y	Y	Y	Y	Y	Y	U	Y	10	90.909091

Result	Paper No	Study	Author	Year	Staffing variable	Staffing Level	Skill	Outcome Variable	Effect	Effect_desc	Not_report	Sig
1	1	1	Betemps et al	1993	Staffing Level	1		Seclusion	Not reported	Not reported	1	0
2	2	2	de Cangas	1993	Staffing Level	1		Seclusion	Not reported	Not reported	1	0
3	3	3	Lanza et al	1994	Staffing Level	1		Aggression	Not reported	Not reported	1	0
4	4	4	Morrison & Lehane	1995	Skill-mix		1	Seclusion	Not reported	Not reported	1	0
5	4	4	Morrison & Lehane	1995	Staffing Level	1		Seclusion	Not reported	Not reported	1	0
6	5	5	Palmstierna & Wistedt	1995	Staffing Level	1		Aggression	Not reported	Not reported	1	0
7	6	6	Lanza et al	1997	Staffing Level	1		Aggression	Not reported	Not reported	1	0
8	7	7	Owen et al	1998	Staffing Level	1		Aggression	Not reported	Not reported	1	0
9	7	7	Owen et al	1998	Skill-mix		1	Aggression	1.35	RR		1
10	8	8	Williams & Myers	2001	Skill-mix		1	Least-restrictive intervention	0.379	r		1
11	9	9	Chou et al	2002	Skill-mix		1	Aggression	0.91	OR		1
12	10	10	Donat	2002	Staffing Level	1		Seclusion	-0.51	r		1
13	11	11	Janssen et al	2007	Staffing Level	1		Seclusion	Not reported	Not reported	1	0
14	11	11	Janssen et al	2007	Skill-mix		1	Seclusion	0.871	OR		1
15	12	12	O'malley et al	2007	Skill-mix		1	Seclusion	NS	F		0
15	12	12	O'malley et al	2007	Staffing Level	1		Seclusion	-0.25	r		1
16	13	13	Pollard et al	2007	Staffing Level	1		Seclusion	Not reported	Not reported	1	0
17	14	14	Bowers et al	2008	Staffing Level	1		Self-Harm	0.94	OR		1
18	15	14	Baker et al	2009	Staffing Level	1		PRN refusal	NS	r		0
19	15	14	Baker et al	2009	Staffing Level	1		Medication refusal	-0.027	r		1
20	15	14	Baker et al	2009	Staffing Level	1		PRN demand	-0.029	r		1
21	16	14	Bowers	2009	Staffing Level	1		Conflict	0.174	r		0
22	16	14	Bowers	2009	Staffing Level	1		Containment	0.125	r		0
23	17	14	Bowers et al	2009	Staffing Level	1		Aggression to others	0.033	r		1
24	18	15	Husum et al	2010	Staffing Level	1		Restraint	Not reported	Not reported	1	0
25	18	15	Husum et al	2010	Staffing Level	1		Sedation	Not reported	Not reported	1	0
26	18	15	Husum et al	2010	Staffing Level	1		Seclusion	Not reported	Not reported	1	0
27	19	14	Bowers & Crowder	2012	Staffing Level	1		Conflict	1.04	RR	1	1
28	19	14	Bowers & Crowder	2012	Staffing Level	1		Containment	1.05	RR		1
29	20	14	Bowers et al	2012	Staffing Level	1		Restraint	0.1	r		0
30	20	14	Bowers et al	2012	Staffing Level	1		Show of force	0.169	r		1
31	21	14	Bowers et al	2013	Staffing Level	1		Conflict & Containmnet	Not reported	Not reported	1	0
32	22	16	Staggs	2013	Skill-mix		1	Aggression	0.94	RR		1
32	22	16	Staggs	2013	Skill-mix		1	Aggression	0.94	RR		1
32	22	16	Staggs	2013	Staffing Level	1		Aggression	1.1	RR		1
32	22	16	Staggs	2013	Staffing Level	1		Aggression	1.1	RR		1
33	23	17	Kalisova et al	2014	Staffing Level	1		Containment	1.05	RR		0
34	24	18	Bak et al (NOR)	2015	Skill-mix		1	Restraint	0.56	RR		0
35	24	18	Bak et al (NOR)	2015	Staffing Level	1		Restraint	0.62	RR		0
36	24	18	Bak et al (DEN)	2015	Staffing Level	1		Restraint	0.96	RR		0
37	24	18	Bak et al (DEN)	2015	Skill-mix		1	Restraint	0.81	RR		0
38	25	19	Staggs	2015	Staffing Level	1		Aggression (patient)	0.81	RR		1
39	25	19	Staggs	2015	Staffing Level	1		Aggression (staff)	1.11	RR		1
40	26	19	Staggs	2016	Staffing Level	1		Aggression	NS	RR		0
41	27	20	Doedens et al	2017	Skill-mix		1	Seclusion	0.98	OR		0
42	27	20	Doedens et al	2017	Staffing Level	1		Seclusion	Not reported	Not reported	1	0
43	28	21	Fukasawa et al	2018	Staffing Level	1		Seclusion	2.36	OR		1

44	28	21	Fukasawa et al	2018	Staffing Level	1		Restraint	1.74	OR		1
45	29	22	Kodal et al.	2018	Staffing Level	1		Restraint	1.11	OR		0
46	30	23	Cook et al	2020	Staffing Level	1		All	Not reported	Not reported	1	0
47	31	24	Park et al	2020	Staffing Level	1		Containment	0.92	OR		1
48	31	24	Park et al	2020	Staffing Level	1		Sedation	1.01	RR		1
49	32	20	Doedens et al	2021	Staffing Level	1		Seclusion	Not reported	Not reported	1	0
50	32	20	Doedens et al	2021	Skill-mix		1	Seclusion	Not reported	Not reported	1	0
51	33	20	Doedens et al	2022	Staffing Level	1		Seclusion	Not reported	Not reported	1	0
52	33	20	Doedens et al	2022	Skill-mix		1	Aggression	Not reported	Not reported	1	0
53	34	25	Rogerson et al	2022	Staffing Level	1		Aggression	2.19	RR		1
54	35	26	Weltens et al	2023	Skill-mix		1	Aggression	3.5	OR		1
						44	14				21	25
